# Potential role of long non‐coding RNA *H19* and *Neat1* in haemophilic arthropathy

**DOI:** 10.1111/jcmm.17770

**Published:** 2023-05-14

**Authors:** Pratiksha Sarangi, Mohankumar B. Senthilkumar, Narendra Kumar, Senthilnathan Senguttuvan, Madavan Vasudevan, Giridhara R. Jayandharan

**Affiliations:** ^1^ Department of Biological Sciences and Bioengineering Indian Institute of Technology Kanpur India; ^2^ Commercial Operations Qiagen India Pvt Ltd. New Delhi India; ^3^ Genomics and Data Science Theomics International Pvt Ltd. Bangalore India

**Keywords:** arthropathy, haemophilia, lncRNA, signalling pathway

Haemophilic arthropathy due to repeated bleeds into the articular cavity is a major cause of morbidity in patients with haemophilia.^1^ While the early phase of haemophilic arthropathy has few similarities with rheumatoid arthritis (RA), such as hyperplasia of the synovium lining, the later stages have more resemblance to osteoarthritis (OA).^2^ However, the underlying mechanism of this disease progression is not well understood. Studies on murine models of haemophilic arthropathy have identified mediators such as Nuclear factor kappa light chain enhancer of activated B cells (NF‐κB) signalling and inflammatory cytokines such as interleukin 1 beta (IL1β) as contributory factors.^3^, ^4^ Recently, the emerging role of long non‐coding RNAs (lncRNAs) in the initiation and progression of chronic inflammatory states such as RA has been reported.^5^, ^6^ Multiple studies have shown dysregulation of lncRNAs such as *H19*, *Neat1* and identified them as potential bio‐markers in collagen induced arthritis (CIA)/OA/RA patients or in animal models.^7^, ^8^, ^9^, ^10^ *H19* is known to be upregulated [~2.75‐fold] in joint tissue and plasma samples of RA patients and is a key player in the activation of inflammatory pathways.^11^ Similarly, *Neat1* is upregulated [~1.5‐fold] in RA and OA joint tissues, which plays an important role in inflammation, chondrocyte apoptosis, and cartilage degradation.^8^, ^12^ Moreover, lncRNAs are key epigenetic regulators of disease pathology,^13^ and can be therapeutic targets^14^ or diagnostic markers^15^ in joint diseases. These data suggest that lncRNAs may also be key molecular mediators of haemophilic arthropathy, a data hitherto not available in the literature.

To test this hypothesis, we first investigated the expression of *H19*, *Neat1* lncRNAs and their target genes in an arthropathy model of haemophilia mice. Haemophilia A mice (F8^tm1kaz/^J, Jackson laboratory) was used to establish a chronic haemophilic arthropathy model as described previously^16^ (Supplementary methods). Total pooled RNA from injured and uninjured joints (*n* = 11 mice) was isolated and converted to cDNA (Qiagen). We then used digital PCR (dPCR) to measure the levels of lncRNAs and protein‐coding genes using QIAcuity digital PCR system (Qiagen). Digital PCR analysis of the joint RNA revealed that lncRNAs *H19* (1727 vs. 446 copies/μL, *p* < 0.001) and *Neat1* (2251 vs. 676 copies/μL, *p* < 0.001) were upregulated in injured joints when compared to control joints (Figure 1A). This finding is consistent with previous reports, where these lncRNAs were dysregulated in affected joint tissues from patients with RA and OA.^8^, ^11^, ^12^, ^17^

**FIGURE 1:**
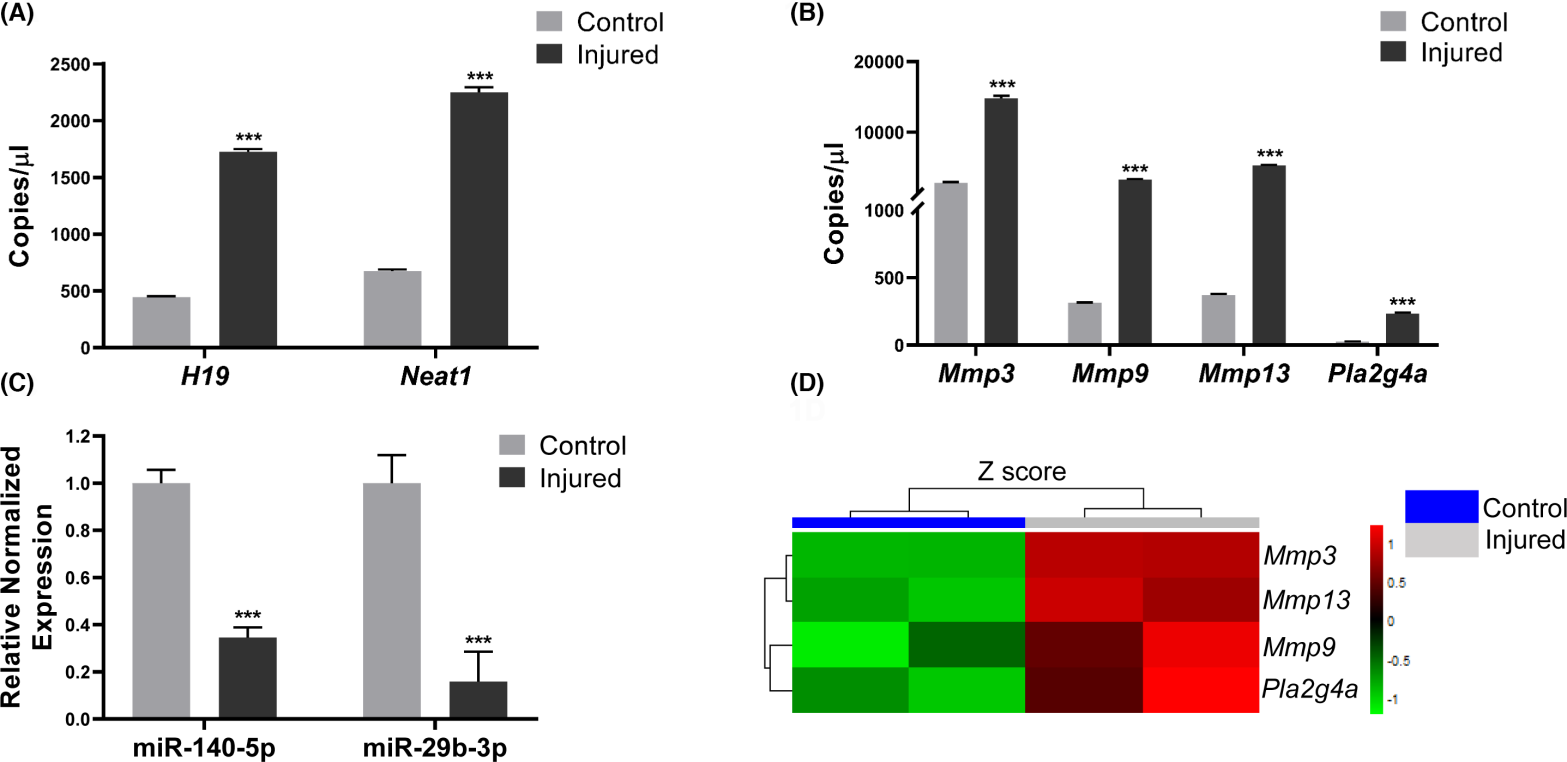
LncRNAs, microRNA and protein coding genes are dysregulated in chronic haemophilic arthropathy. Total pooled RNA from control and injured knee joints (*n* = 11 mice) was converted to cDNA and the absolute expression of target genes was analysed by digital PCR (please refer supplementary methods). The absolute concentration of lncRNAs (*n* = 3 replicates) (A), target protein coding genes (*n* = 3 replicates) (B) are shown. The data represent mean ± SD. Relative quantification of microRNA (*n* = 6 replicates) was performed by quantitative PCR. Representative data are shown for relative normalized expression (C). U6 snRNA was used as a control for normalization of microRNA expression. Differential gene expression for the target genes obtained independently from injured and control joints of haemophilic mice (*n* = 8) are plotted from the transcriptome sequencing data (CuffDiff, Illumina NovaSeq 6000) obtained from injured and control joints (D) (***p < 0.001 vs. control joint).

We then wished to understand if the lncRNAs alter the expression of specific protein coding genes in haemophilic arthropathy. LncRNA *H19*, which regulates the expression of matrix metalloproteinases (*Mmp9* and *Mmp13*)^10^, ^18^ is known to play a key role in other joint diseases such as OA by increasing the levels of MMPs (by ~2.2‐fold) and leading to cartilage degeneration.^19^ An absolute quantification of these targets in the chronic arthropathy model, demonstrated elevated *Mmp3* (14,788 vs. 2846 copies/μL), *Mmp9* (3331 vs. 314 copies/μL) and *Mmp13* (5278 vs. 370 copies/μL) in the injured joints (Figure 1B). To further understand the mechanism by which MMPs are regulated by *H19* lncRNA, we profiled the levels of microRNAs (miR‐140‐5p and miR‐29b‐3p) that are key intermediates in tuning MMP9 and MMP13 expression.^10^, ^18^ The potential binding of these microRNAs to the target sequences were verified using computational approach (please refer Supplementary Methods). The seed sequence of mmu‐miR‐29b‐5p (5′‐UAGCACCAUUU‐3′) binds to the target sequence of *H19*. Similarly, the seed sequence of mmu‐miR‐140‐5p (5′‐AGUGGUU‐3′) binds to *H19* RNA sequence. A relative estimation of miR‐140‐5p and miR‐29b‐3p in joint RNA by quantitative PCR showed reduced expression (−2.89‐fold and − 6.31‐fold, respectively) in injured joints (Figure 1C), indicating a pivotal role of lncRNA *H19*‐ miR‐140‐5p and miR‐29b‐3p axis in haemophilic arthropathy. Similarly, lncRNA *Neat1* is known to alter the cytosolic phospholipase A2 (cPLA2), an enzyme encoded by *Pla2g4a* by sequestering miR‐543.^12^ The binding of mmu‐miR‐543‐3p seed sequence (5′‐AACAUUC‐3′) to the target lncRNA *Neat1* and *Pla2g4a* was confirmed by computational analysis. Interestingly, in our analysis, *Pla2g4a* was highly expressed (233 vs. 26 copies/μL, *p* < 0.001) in the injured joints (Figure 1B) in haemophilic arthropathy. This suggests that lncRNA *Neat1* and *Pla2g4a* are positive regulators of *Mmp3* ^12^, ^20^ and *Mmp13* activity as described earlier in a collagen induced arthritis mice model.^21^

To further validate these findings, we next performed an independent study with mRNA‐based gene expression profiling from total joint RNA of arthropathy model (*n* = 8 mice) (Illumina NovaSeq 6000). As can be seen in Figure 1D, the levels of *Mmp3*, *Mmp9*, *Mmp13* were significantly upregulated (*p* < 0.05) in the injured joints. The levels of *Pla2g4a* were elevated in the injured joints but the results were not statistically significant (*p* = 0.08). To corroborate these findings, we further investigated the levels of target proteins in the haemarthritic joints by immunohistochemistry (*n* = 3 mice per antibody; total mice used, *n* = 7). The clinical features of haemophilic arthropathy were first confirmed by haematoxylin and eosin staining wherein inflammation of the injured joint was observed with degeneration of the articular cartilage (Figure 2A). Consistent with the gene expression data, the levels of MMPs 3, 9 and 13, and cPLA2 were higher and sequestered in the articular cartilage of injured joints (Figure 2B,C). This highlights their degenerative role on the extracellular matrix of the cartilage tissue, leading to haemophilic arthropathy.

**FIGURE 2:**
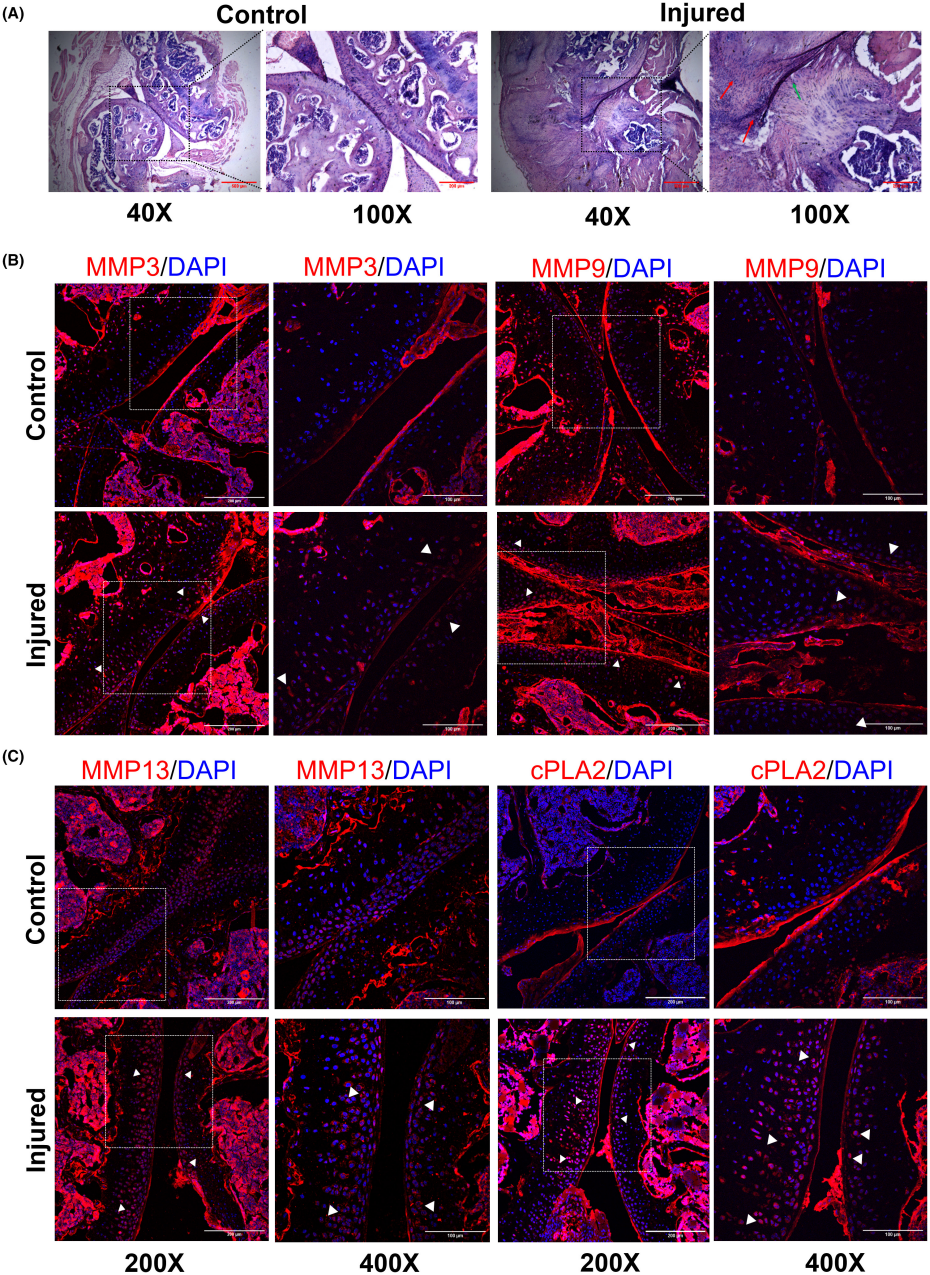
Morphological and immunohistochemical analysis validates the dysregulation of matrix metalloproteinases (MMPs) and cytosolic phospholipase A2 (cPLA2) in the joints of haemophilic arthropathy mice. Joint tissues were harvested from haemophilia A mice (*n* = 7) and fixed in 4% paraformaldehyde and decalcified in 14% EDTA for 14 days. Cryosections of joint tissue; 8 μm section thickness (Leica CM1520, Leica Biosystems) were obtained from injured and control joints. Haematoxylin and eosin staining shows swelling and inflammation in the injured joint (A), synovial hyperplasia (indicated by red arrows) and cartilage erosion (indicated by green arrow). Scale bar is 500 μm (for 40×); 200 μm (for 100×). These sections (*n* = 3 mice) were further stained with primary antibodies to the MMPs 3, 9, 13 and cPLA2 (B, C) (please refer supplementary methods). Our data reveals elevated expression of these molecular regulators in injured joints (denoted by white arrows). The secondary antibody control staining demonstrated negligible expression of target proteins (data not shown). Images were obtained by confocal microscopy (Zeiss LSM780, Carl Zeiss AG). Scale bar is 100 μm (for 400×); 200 μm (for 200×). High magnification images of the articular cartilage are depicted from the region marked in lower magnification.

The upregulation of the lncRNAs *H19* and *Neat1* in injured joints is consistent with previous findings in patients with other joint diseases such as RA and OA.^11^, ^14^, ^15^, ^19^ This altered lncRNA expression leads to an imbalance in the regulatory nodes involving miRNAs or enzymatic mediators such as cytosolic phospholipase A2 that regulate MMP synthesis, thereby influencing disease manifestation and progression.^22^ Interestingly, these lncRNAs have also been extensively investigated for their role in multiple cancer types,^23^ suggesting their link to the proliferative phenotype seen in synovial hyperplasia and cartilage turnover. Nonetheless, the pathobiology of haemophilic arthropathy is primarily influenced by blood induced joint inflammation and cartilage degradation,^24^ and a detailed spatio‐temporal profiling may be necessary to completely understand the effect of lncRNA deregulation and its hitherto unknown roles in joint disease. Since many of these lncRNAs and associated pathways are conserved,^25^ this knowledge can benefit a variety of other joint diseases such as RA and OA.

In conclusion, we report here that lncRNA *H19* and *Neat1* are highly expressed in a murine model of chronic haemophilic arthropathy. This is the first study to document the role of lncRNAs in the development of haemophilic arthropathy (Figure S1). Based on our data, we reason that these lncRNAs play a regulatory role in the development of arthropathy via MMPs 3, 9 and 13 that have been previously implicated in haemophilic joint disease.^3^, ^4^ Further studies are warranted, to understand the mechanistic basis of this regulatory axis (lncRNA/microRNA/protein‐coding genes) and their potential as therapeutic targets to attenuate the progression of joint disease.

## AUTHOR CONTRIBUTIONS

**Pratiksha Sarangi:** Investigation (lead); methodology (lead). **Mohankumar B. Senthilkumar:** Formal analysis (supporting); investigation (lead); methodology (supporting). **Narendra kumar:** Formal analysis (equal); investigation (equal); methodology (equal). **SenthilNathan Senguttuvan:** Formal analysis (equal); investigation (equal); methodology (equal). **V Madavan:** Data curation (equal); formal analysis (equal); software (lead). **Giridhara R. Jayandharan:** Conceptualization (lead); funding acquisition (lead); methodology (supporting); project administration (lead).

## CONFLICT OF INTEREST STATEMENT

The authors (GRJ, PS, MK, NK) have applied for patents on AAV technology for gene therapy and some of them have been licensed to commercial entities.

## Supporting information


**Appendix S1:** Supporting Information

## Data Availability

The data that support the findings of this study are available from the corresponding author upon reasonable request.
