# Supplementary material for: Potential role of long non‐coding RNA H19 and Neat1 in haemophilic arthropathy
Source: J Cell Mol Med. 2023 May 14;27(12):1745–9. doi: 10.1111/jcmm.17770 (PMC10273061; doi:10.1111/jcmm.17770)
Supplement: Supplementary file 1 — Appendix S1: Supporting Information [file JCMM-27-1745-s001.docx]

**Supplementary methods:**

**1.** **Chronic hemophilic arthropathy model**

To generate a model of chronic hemophilic arthropathy (HA), multiple bleeding episodes on Day 0, 14 and 30 were induced by a sharp injury in the right knee of hemophilia A mice (n=32) of 8 to 14 weeks of age as described earlier.^1^ The contralateral uninjured left knee joint served as a control tissue. Multiple injuries during the model-development process resulted in mortality which has been reported in the previous studies.^2^ Subsequently samples were harvested on Day31 from mice that survived multiple injuries / bleeding episodes.

**2. RNA isolation**

For RNA based studies, injured (right knee) joints and uninjured (contralateral left knee) joints were pooled in two batches (n=11 and n=8 mice), respectively, for RNA extraction. The joint tissue was collected by excising at the distal end of femur and proximal end of tibia in RNA protect reagent (Qiagen, Hilden, Germany). The muscle tissue and synovium around the joint were removed and the articular cartilage was collected from femoral condyles and tibial plateau. Pooled RNA was isolated from cartilage tissue using Trizol method. Of these, total RNA (batch 1: n=11) was converted to cDNA using Quantitect reverse transcription kit (Qiagen) for quantification of lncRNAs, microRNAs and target genes. Pooled RNA from control and injured joints (batch 2: n=8) was used for global mRNA sequencing.

**3. Quantification of lncRNAs and target genes by digital PCR**

The lncRNAs and protein-coding genes were quantified using QIAcuity digital (d) PCR system (Qiagen). The reaction mix of 12ul was prepared using 400nM of primer mix, 1X Evagreen PCR master-mix, 75ng of cDNA, and nuclease free water. The reaction mix was loaded into 8.5K nanoplate in triplicates for each sample and dPCR was performed. The dPCR involves three major steps: (1) Nanowell generation (8.5K nanowells for each reaction mix), (2) PCR amplification of targets, and (3) detection of positive and negative fluorescence signal. The dPCR conditions were as follows: initial denaturation of 2 minutes at 95°C, followed by 40 cycles of 15 seconds at 95°C, 15 seconds at 58°C, 15 seconds at 72°C and cooling of 5 minutes at 40°C. Each target was analyzed in triplicates.

**4.** **Computational based prediction of microRNA target binding sites**

The microRNA sequences for mmu-miR-29b-3p, mmu-miR-140-5p and mmu-miR-543-3p were retrieved from miRBase.^3^ The binding of microRNAs to the target lncRNAs *H19* and *Neat1* was confirmed by IntaRNA2.0, an RNA-RNA interaction prediction tool.^4^ Additionally, the microRNA-protein target binding for *Pla2g4a* was predicted using TargetScan8.0.^5^

**5. Estimation of microRNAs in joint tissues**

Total RNA from control and injured joint tissue (n=11) was used to convert into cDNA using miRCURY LNA RT kit (Qiagen). Relative expression of miR-29b-3p and miR-140-5p was measured using 12ng of template, 100nM forward and reverse primers (Qiagen), 2X GoTaq qPCR master-mix (Promega), and nuclease free water as per the manufacturer’s protocol. PCR cycling conditions were as follows: initial denaturation of 2 minutes at 95°C, followed by 40 cycles of annealing and extension at 56°C for 1 minute. U6snRNA was used as reference to normalize microRNA expression across different conditions.

**6. Global mRNA sequencing in chronic hemoarthritic joints**

Pooled RNA (n=8 mice) was checked for its integrity by Agilent Bioanalyzer 2100 and samples with clean rRNA peaks were used for further investigation. Libraries for RNA-seq were prepared according to KAPA stranded RNA-seq kit with RiboErase (KAPA Biosystems, Wilmington, MA, USA) system. Final library quality and quantity were analyzed by Agilent Bioanalyzer 2100 and Life Technologies Qubit3.0 Fluorometer, respectively. A 150 bp paired-end sequencing was performed on Illumina NovoSeq 6000 (Illumnia Inc., San Diego, CA, USA). Mouse genome (Mm10) was downloaded from iGenomes and indexed using Bowtie2-build with default parameters. Adapter removal was done using Trim Galore (v 0.4.4) and each of the raw Fastq files were passed through a quality check using FastQC. PCR duplicates were removed using the Samtools 1.3.1 with the help of ‘rmdup’ option. Each of the raw files was then aligned to mm10 genome assembly using TopHat2 with default parameters for paired-end sequencing. After aligning, quantification of transcripts was performed using Cufflinks, and then Cuffmerge was used to create merged transcriptome annotation. Replicate reproducibility was ascertained using PCA plot and unsupervised hierarchical condition tree. Finally, differentially expressed (DE) genes were identified using Cuffdiff. The threshold for DE genes was log2 (fold change) >1.5 for up-regulated genes and log2 (fold change) <1.5 for down-regulated genes with *p value <0.05*.

**7. Immunohistochemistry**

After joint injury on Day0, Day14 and Day30 to induce chronic hemophilic arthropathy, knee-joints (n=7) were collected on Day31. After isolation, the knee joints were fixed in 4% paraformaldehyde (PFA) for 48 hrs at 4°C. Joint tissues were decalcified in 14% ethylene diamine tetra acetic acid (EDTA) for 14 days. Cryosections were obtained from the decalcified joint tissues using polyfreeze (Sigma Aldrich, St. Louis, Missouri, United States). Tissue sections were fixed with 4% PFA and washed with phosphate buffered saline with 1% Tween 20 (PBST). The sections (n=3 mice per antibody) were probed with primary antibodies to MMP3 (1:50, sc-21732), MMP9 (1:100, sc-393859), cPLA2 (1:100, sc-454) (Santacruz Biotechnology, Santacruz, California, USA), and MMP13 (1:100, ab39012) (Abcam, Cambridge, United Kingdom) for 24 hrs at 4°C. After washing with PBST, the sections were subjected to blocking with normal goat serum (Abcam) for overnight at 4°C. Further, incubation with secondary antibody, goat anti-mouse Alexafluor 568 (1:500, A-11004) (Invitrogen, Waltham, Massachusetts, USA) or goat anti-rabbit Cy3 (1:200, 111-165-008) (Jackson Immuno Research, West Grove, Pennsylvania, USA) was performed at room temperature for 1 hr. After washing with PBST, 4′,6-diamidino-2-phenylindole (DAPI) staining (Sigma Aldrich) was performed. Mounting was done using Fluorsave (Merck, Darmstadt, Germany) and kept in 4°C until imaging. For secondary control staining, the sections were blocked with normal goat serum followed by incubation with secondary antibody (data not shown). Further, they were counter stained with DAPI and mounted before imaging. Confocal imaging (LSM780NLO, Carl Zeiss GmbH, Wein, Austria) was performed to visualize the target proteins.

**8. Data analysis**

The absolute copies/µl for the gene of interest was exported from the QIAcuity software and used for data analysis. Data are represented as mean + SD. An unpaired Student’s t-test was performed to compare the gene expression between control and injured group. All the graphs were rendered using GraphPad Prism 8.0.2 and quantitative PCR was analysed by Biorad CFX Manager 3.1. Fisher's exact test was performed for differential gene expression analysis with an FDR of <0.05. A p-value < 0.05 was considered to be statistically significant between the control and injured joints.


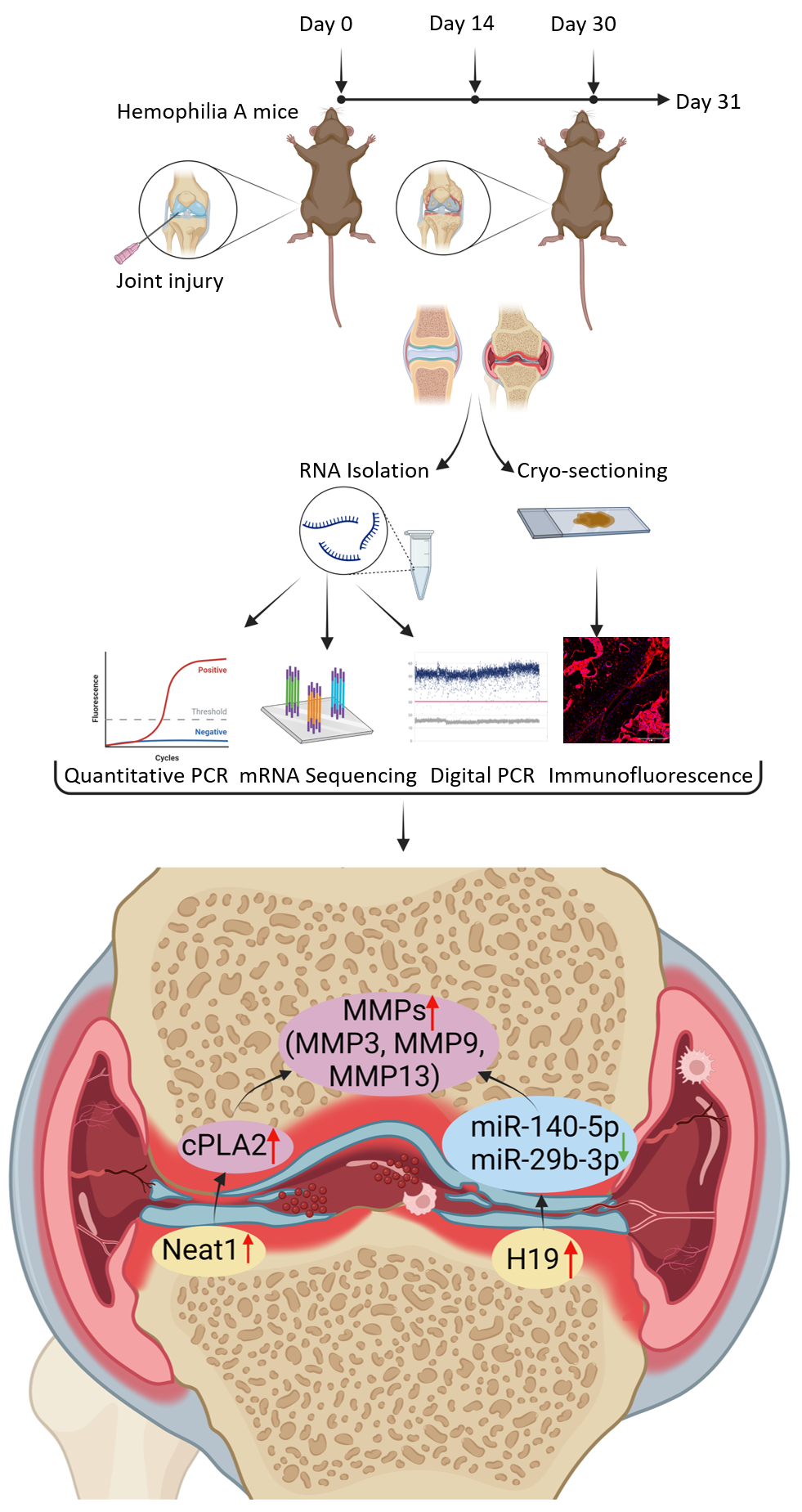


**Supplementary Figure 1: LncRNAs *H19* and *Neat1* regulate MMPs in a murine model of hemophilic arthropathy.** Recurrent bleeding into the joints leads to accumulation of hemosiderin which is a key player in induction of inflammation in the joints subsequently followed by degradation of cartilage and bone.^6,7^ The pathogenesis of HA involves various molecular mediators dictating the fate of this joint disease.^2,8^ In the present study, lncRNA *H19* was found to be upregulated in injured joints, which then positively regulates MMP expression *via* sequestration of miRNAs, miR-140-5p and miR-29b-3p. Another crucial lncRNA, *Neat1*, was also observed to be elevated in the injured joints, leading to over expression of MMP3 and MMP13 *via* cPLA2 mediator. The abundance of MMPs possibly disrupts the cartilage and results in irreversible hemophilic joint disease.

**References:**

1. Hakobyan N, Enockson C, Cole AA, Sumner DR, Valentino LA. Experimental haemophilic arthropathy in a mouse model of a massive haemarthrosis: gross, radiological and histological changes. *Haemophilia.* 2008;14(4):804-809.

2. Sen D, Chapla A, Walter N, Daniel V, Srivastava A, Jayandharan GR. Nuclear factor (NF)‐κ B and its associated pathways are major molecular regulators of blood‐induced joint damage in a murine model of hemophilia. *J Thromb Haemost.* 2013;11(2):293-306.

3. Kozomara A, Birgaoanu M, Griffiths-Jones S. miRBase: from microRNA sequences to function. *Nucleic Acids Res*. 2019;47(D1):D155-62.

4. Mann M, Wright PR, Backofen R. IntaRNA 2.0: enhanced and customizable prediction of RNA–RNA interactions. *Nucleic Acids Res*. 2017;45(W1):W435-9.

5. McGeary SE, Lin KS, Shi CY, et al. The biochemical basis of microRNA targeting efficacy. *Science*. 2019;366(6472):eaav1741.

6. Mignot S, Rothschild C, Harroche A, et al. Unique inflammatory signature in hemophilic arthropathy: Epigenetic changes due to interaction between blood and fibroblast-like synoviocytes. *Blood.* 2017;130:3669.

7. Gualtierotti R, Solimeno LP, Peyvandi F. Hemophilic arthropathy: current knowledge and future perspectives. *J Thromb Haemost.* 2021;19(9):2112-2121.

8. Sen D, Jayandharan GR. MicroRNA-15b modulates molecular mediators of blood induced arthropathy in hemophilia mice. *Int J Mol Sci.* 2016 Apr 8;17(4):492.
